# Supplementary material for: Ability and accuracy of the smartphone-based O`VIEW-M® sperm test: Useful tool in the era of Covid-19
Source: PLoS One. 2022 Jun 16;17(6):e0269894. doi: 10.1371/journal.pone.0269894 (PMC9202868; doi:10.1371/journal.pone.0269894)
Supplement: S1 File — (DOCX) [file pone.0269894.s001.docx]

## **Validity Evaluation (Accuracy Evaluation)**

| CODE | OPTION | | A | B | A-1 | B-1 |
| --- | --- | --- | --- | --- | --- | --- |
|  | vasectomy | 5 days of abstinence | sperm count (million) | OVIEW-M  sperm count | motility (%) | OVIEW-M motility |
| A-01-001 | y | y | 0 | 0 | 0 | 0 |
| A-01-002 |  | y | 20 | 23 | 40 | 43 |
| A-01-003 |  | y | 25 | 27 | 40 | 42 |
| A-01-004 | y | y | 0 | 0 | 0 | 0 |
| A-01-005 | y | y | 0 | 0 | 0 | 0 |
| A-01-006 |  | y | 20 | 19 | 30 | 33 |
| A-01-007 |  | y | 35 | 39 | 40 | 42 |
| A-01-008 |  | y | 45 | 46 | 50 | 48 |
| A-01-009 |  | y | 20 | 19 | 40 | 41 |
| A-01-010 | y | y | 0 | 0 | 0 | 0 |
| A-02-011 |  | y | 55 | 55 | 60 | 58 |
| A-02-012 |  | y | 70 | 65 | 70 | 71 |
| A-02-013 |  | y | 10 | 10 | 20 | 18 |
| A-02-014 |  | y | 25 | 20 | 40 | 37 |
| A-02-015 | y | y | 0 | 0 | 0 | 0 |
| A-02-016 |  | y | 20 | 20 | 30 | 28 |
| A-02-017 |  | y | 55 | 50 | 60 | 59 |
| A-02-018 | y | y | 0 | 0 | 0 | 0 |
| A-02-019 |  | y | 5 | 5 | 20 | 16 |
| A-02-020 |  | y | 25 | 27 | 40 | 36 |
| A-03-021 |  | y | 45 | 41 | 50 | 48 |
| A-03-022 | y | y | 0 | 0 | 0 | 0 |
| A-03-023 |  | y | 35 | 31 | 40 | 37 |
| A-03-024 |  | y | 75 | 71 | 80 | 84 |
| A-03-025 |  | y | 20 | 22 | 40 | 37 |
| A-03-026 | y | y | 0 | 0 | 0 | 0 |
| A-03-027 |  | y | 10 | 10 | 30 | 29 |
| A-03-028 |  | y | 45 | 42 | 70 | 64 |
| A-03-029 | y | y | 0 | 0 | 0 | 0 |
| A-03-030 |  | y | 40 | 41 | 40 | 40 |
| A-04-031 |  | y | 80 | 84 | 60 | 65 |
| A-04-032 |  | y | 10 | 10 | 10 | 0 |
| A-04-033 | y | y | 0 | 0 | 0 | 0 |
| A-04-034 |  | y | 20 | 20 | 30 | 28 |
| A-04-035 | y | y | 0 | 0 | 0 | 0 |
| A-04-036 |  | y | 95 | 93 | 90 | 84 |
| A-04-037 |  | y | 25 | 22 | 40 | 43 |
| A-04-038 | y | y | 0 | 0 | 0 | 0 |
| A-04-039 |  | y | 40 | 38 | 70 | 69 |
| A-04-040 | y | y | 0 | 0 | 0 | 0 |
| A-05-041 |  | y | 30 | 30 | 50 | 47 |
| A-05-042 |  | y | 20 | 19 | 30 | 28 |
| A-05-043 | y | y | 0 | 0 | 0 | 0 |
| A-05-044 |  | y | 70 | 72 | 70 | 70 |
| A-05-045 |  | y | 30 | 29 | 40 | 37 |
| A-05-046 | y | y | 0 | 0 | 0 | 0 |
| A-05-047 |  | y | 30 | 30 | 30 | 32 |
| A-05-048 |  | y | 60 | 59 | 60 | 64 |
| A-05-049 | y | y | 0 | 0 | 0 | 0 |
| A-05-050 |  | y | 40 | 35 | 50 | 54 |
| A-05-051 |  | y | 20 | 17 | 40 | 44 |
| A-05-052 |  | y | 35 | 35 | 70 | 78 |
| A-05-053 |  | y | 40 | 35 | 60 | 55 |
| A-05-054 | y | y | 0 | 0 | 0 | 0 |
| A-05-055 |  | y | 15 | 20 | 30 | 34 |
| A-05-056 |  | y | 75 | 76 | 70 | 75 |
| A-05-057 |  | y | 70 | 68 | 70 | 74 |
| A-05-058 |  | y | 50 | 54 | 60 | 58 |
| A-05-059 |  | y | 65 | 66 | 80 | 78 |
| A-05-060 | y | y | 0 | 0 | 0 | 0 |
| A-06-061 |  | y | 45 | 46 | 60 | 58 |
| A-06-062 |  | y | 50 | 51 | 70 | 68 |
| A-06-063 |  | y | 25 | 20 | 40 | 43 |
| A-06-064 |  | y | 50 | 48 | 50 | 48 |
| A-06-065 |  | y | 65 | 67 | 70 | 68 |
| A-06-066 |  | y | 70 | 74 | 60 | 59 |
| A-06-067 | y | y | 0 | 0 | 0 | 0 |
| A-06-068 |  | y | 85 | 81 | 70 | 68 |
| A-06-069 |  | y | 20 | 20 | 40 | 38 |
| A-06-070 |  | y | 55 | 55 | 60 | 54 |
| A-06-071 | y | y | 0 | 0 | 0 | 0 |
| A-06-072 |  | y | 25 | 23 | 30 | 29 |
| A-06-073 |  | y | 30 | 35 | 40 | 42 |
| A-06-074 |  | y | 40 | 36 | 50 | 53 |
| A-06-075 |  | y | 65 | 67 | 70 | 73 |
| A-06-076 |  | y | 55 | 54 | 60 | 59 |
| A-06-077 |  | y | 80 | 80 | 80 | 78 |
| A-06-078 |  | y | 65 | 64 | 60 | 62 |
| A-06-079 |  | y | 60 | 62 | 50 | 51 |
| A-06-080 | y | y | 0 | 0 | 0 | 0 |
| A-07-081 |  | y | 70 | 73 | 60 | 56 |
| A-07-082 |  | y | 65 | 63 | 70 | 73 |
| A-07-083 |  | y | 45 | 46 | 60 | 68 |
| A-07-084 |  | y | 30 | 30 | 30 | 29 |
| A-07-085 |  | y | 40 | 39 | 50 | 51 |
| A-07-086 |  | y | 20 | 20 | 40 | 43 |
| A-07-087 |  | y | 95 | 93 | 80 | 87 |
| A-07-088 |  | y | 90 | 91 | 70 | 72 |
| A-07-089 |  | y | 80 | 85 | 60 | 59 |
| A-07-090 | y | y | 0 | 0 | 0 | 0 |
| A-07-091 |  | y | 75 | 71 | 50 | 45 |
| A-07-092 |  | y | 90 | 88 | 70 | 68 |
| A-07-093 |  | y | 75 | 76 | 60 | 61 |
| A-07-094 | y | y | 0 | 0 | 0 | 0 |
| A-07-095 |  | y | 80 | 78 | 70 | 69 |
| A-07-096 |  | y | 30 | 30 | 40 | 41 |
| A-07-097 |  | y | 85 | 80 | 70 | 72 |
| A-07-098 |  | y | 90 | 90 | 60 | 59 |
| A-07-099 |  | y | 100 | 102 | 80 | 86 |
| A-07-100 |  | y | 25 | 24 | 50 | 47 |
| A-08-101 | y | y | 0 | 0 | 0 | 0 |
| A-08-102 |  | y | 80 | 83 | 70 | 68 |
| A-08-103 |  | y | 70 | 71 | 70 | 69 |
| A-08-104 |  | y | 60 | 59 | 60 | 57 |
| A-08-105 |  | y | 30 | 31 | 50 | 58 |
| A-08-106 |  | y | 55 | 55 | 70 | 69 |
| A-08-107 |  | y | 50 | 45 | 50 | 51 |
| A-08-108 | y | y | 0 | 0 | 0 | 0 |
| A-08-109 |  | y | 45 | 45 | 60 | 63 |
| A-08-110 |  | y | 35 | 39 | 50 | 54 |
| A-08-111 |  | y | 25 | 27 | 30 | 31 |
| A-08-112 |  | y | 25 | 24 | 40 | 41 |
| A-08-113 |  | y | 55 | 59 | 70 | 70 |
| A-08-114 |  | y | 40 | 40 | 60 | 59 |
| A-08-115 |  | y | 40 | 41 | 50 | 49 |
| A-08-116 |  | y | 30 | 30 | 40 | 43 |
| A-08-117 | y | y | 0 | 0 | 0 | 0 |
| A-08-118 |  | y | 110 | 112 | 70 | 68 |
| A-08-119 |  | y | 80 | 78 | 60 | 58 |
| A-08-120 |  | y | 95 | 94 | 80 | 79 |
| A-09-121 |  | y | 65 | 68 | 60 | 61 |
| A-09-122 |  | y | 55 | 57 | 50 | 49 |
| A-09-123 | y | y | 0 | 0 | 0 | 0 |
| A-09-124 |  | y | 80 | 81 | 70 | 69 |
| A-09-125 |  | y | 30 | 32 | 60 | 58 |
| A-09-126 |  | y | 45 | 47 | 50 | 51 |
| A-09-127 |  | y | 40 | 40 | 40 | 39 |
| A-09-128 |  | y | 80 | 78 | 80 | 79 |
| A-09-129 |  | y | 65 | 69 | 60 | 59 |
| A-09-130 |  | y | 55 | 57 | 50 | 48 |
| A-09-131 |  | y | 30 | 30 | 40 | 40 |
| A-09-132 |  | y | 70 | 73 | 60 | 59 |
| A-09-133 | y | y | 0 | 0 | 0 | 0 |
| A-09-134 |  | y | 85 | 82 | 80 | 79 |
| A-09-135 |  | y | 85 | 86 | 70 | 75 |
| A-09-136 |  | y | 65 | 63 | 50 | 54 |
| A-09-137 |  | y | 70 | 67 | 60 | 63 |
| A-09-138 |  | y | 80 | 78 | 70 | 71 |
| A-09-139 |  | y | 70 | 71 | 60 | 58 |
| A-09-140 |  | y | 35 | 37 | 50 | 49 |
| A-10-141 |  | y | 25 | 27 | 40 | 39 |
| A-10-142 |  | y | 85 | 85 | 70 | 69 |
| A-10-143 |  | y | 85 | 88 | 80 | 77 |
| A-10-144 |  | y | 65 | 65 | 60 | 59 |
| A-10-145 |  | y | 70 | 74 | 70 | 68 |
| A-10-146 |  | y | 40 | 35 | 50 | 52 |
| A-10-147 |  | y | 35 | 37 | 40 | 41 |
| A-10-148 |  | y | 65 | 64 | 70 | 78 |
| A-10-149 |  | y | 60 | 55 | 60 | 55 |
| A-10-150 |  | y | 30 | 32 | 40 | 43 |
| A-10-151 |  | y | 55 | 59 | 50 | 51 |
| A-10-152 |  | y | 70 | 69 | 70 | 75 |
| A-10-153 |  | y | 80 | 82 | 60 | 57 |
| A-10-154 |  | y | 105 | 106 | 70 | 71 |
| A-10-155 |  | y | 85 | 80 | 60 | 58 |
| A-10-156 |  | y | 80 | 80 | 50 | 48 |
| A-10-157 |  | y | 90 | 91 | 70 | 68 |
| A-10-158 |  | y | 80 | 75 | 60 | 59 |
| A-10-159 |  | y | 95 | 95 | 80 | 78 |
| A-10-160 |  | y | 90 | 91 | 70 | 77 |
| A-11-161 |  | y | 80 | 79 | 60 | 56 |
| A-11-162 |  | y | 90 | 95 | 80 | 79 |
| A-11-163 |  | y | 80 | 81 | 70 | 69 |
| A-11-164 |  | y | 65 | 63 | 50 | 51 |
| A-11-165 |  | y | 75 | 76 | 70 | 73 |
| A-11-166 |  | y | 25 | 25 | 40 | 42 |
| A-11-167 |  | y | 90 | 90 | 80 | 77 |
| A-11-168 |  | y | 80 | 83 | 60 | 64 |
| A-11-169 |  | y | 75 | 76 | 70 | 67 |
| A-11-170 |  | y | 70 | 74 | 70 | 75 |
| A-11-171 |  | y | 65 | 63 | 60 | 58 |
| A-11-172 |  | y | 50 | 49 | 50 | 46 |
| A-11-173 |  | y | 60 | 62 | 60 | 67 |
| A-11-174 |  | y | 85 | 84 | 70 | 72 |
| A-11-175 |  | y | 105 | 107 | 80 | 82 |
| A-11-176 |  | y | 30 | 30 | 40 | 43 |
| A-11-177 |  | y | 55 | 55 | 60 | 63 |
| A-11-178 |  | y | 60 | 57 | 70 | 71 |
| A-11-179 |  | y | 45 | 47 | 80 | 75 |
| A-11-180 |  | y | 35 | 34 | 50 | 54 |
| A-12-181 |  | y | 35 | 36 | 60 | 64 |
| A-12-182 |  | y | 85 | 86 | 70 | 72 |
| A-12-183 |  | y | 55 | 51 | 60 | 59 |
| A-12-184 |  | y | 35 | 33 | 40 | 43 |
| A-12-185 |  | y | 60 | 60 | 60 | 63 |
| A-12-186 |  | y | 95 | 95 | 70 | 76 |
| A-12-187 |  | y | 70 | 68 | 50 | 54 |
| A-12-188 |  | y | 80 | 75 | 60 | 62 |
| A-13-189 |  | y | 80 | 80 | 70 | 73 |
| A-13-190 |  | y | 110 | 109 | 80 | 77 |
| A-13-191 |  | y | 95 | 94 | 70 | 68 |
| A-13-192 |  | y | 90 | 87 | 60 | 59 |
| A-13-193 |  | y | 45 | 44 | 70 | 69 |
| A-13-194 |  | y | 40 | 41 | 60 | 59 |
| A-13-195 |  | y | 115 | 113 | 80 | 78 |
| A-13-196 |  | y | 45 | 44 | 50 | 49 |
| A-13-197 |  | y | 90 | 88 | 60 | 58 |
| A-14-198 |  | y | 85 | 86 | 70 | 68 |
| A-14-199 |  | y | 100 | 99 | 80 | 78 |
| A-14-200 |  | y | 80 | 83 | 70 | 68 |
