# Supplementary material for: Ability and accuracy of the smartphone-based O`VIEW-M® sperm test: Useful tool in the era of Covid-19
Source: PLoS One. 2022 Jun 16;17(6):e0269894. doi: 10.1371/journal.pone.0269894 (PMC9202868; doi:10.1371/journal.pone.0269894)
Supplement: Attachment — Submitted filename: Revisionl letter_ver.4.doc [file pone.0269894.s002.doc]

**Revision Letter**

Dear Editor, Edrian Nim Tolentino

We are pleased to submit to you a revised version of our manuscript #: PONE-D-21-36708, Title: "Ability and Accuracy of the Smartphone-based O`VIEW-M® Sperm Test: Useful tool in the era of Covid-19.” with hope of it being published in PLOS ONE. Followings are comments on our manuscript along with our replies.

Sincerely,

Corresponding author

- The followings are our responses to the queries given by Reviewers.

1. We note that you provided the following response to our query regarding any patents or products in development related to this research: "Yes. The products in development or marketed products associated with this research are as follows. A measuring device of salive for smart phone (Reg. No. 10-1533343), The test device for Body fluid analysis using natural light (Reg. No. 10-1813866), CHAMBER FOR INSPECTING BODY-FLUID AND BODY-FLUID INSPECTING APPARATUS USING THE SAME (Reg. No. 10-0029588) and so on."

Please clarify what is meant by "and so on", and whether there are any additional patents, products in development or marketed products associated with this research to declare that were not included in your response above.

“Yes. As you mentioned, we have confirmed that there are no additional patents, products in development or products on the market related to this research. Therefore, there are A measuring device of salive for smart phone (Reg. No. 10-1533343), The test device for Body fluid analysis using natural light (Reg. No. 10-1813866), CHAMBER FOR INSPECTING BODY-FLUID AND BODY-FLUID INSPECTING APPARATUS USING THE SAME (Reg. No. 10-0029588) products related to this research.”

2. Please review our data sharing policy: https://journals.plos.org/plosone/s/data-availability

PLOS journals require authors to make all data necessary to replicate their study’s findings publicly available without restriction at the time of publication. When specific legal or ethical restrictions prohibit public sharing of a data set, authors must indicate how others may obtain access to the data.

“Yes. We submit the raw data used in this study as a supplement to indicate how others may obtain access to the data.”
